# The Student in Vienna

**Published:** 1892-09-24

**Authors:** 


### The Student in Vienna.

"He has studied in Vienna" one often hears it said of some young medical man. A few of the young man's friends may know something of his experiences there, but to the general public the expression conveys only vague and confused ideas of bearded and spectacled professors, vivisection, cigars, and beer. But as about two hundred English-speaking medical men, mostly newly qualified, visit Vienna each year, and remain there on an average three or four months, some account of how they live and what they do may be of interest.

Let us suppose, then, that Robert Sawyer, B.A., M.B., &c.—a better-qualified and harder-working man than his grandfather was—has arrived in Wien. With the help of some college friend there before him, or of that most useful body, the "Anglo-American Wiener Medical Association, he has secured rooms near the hospital. He talks of "rooms," but, as a matter of fact, no one thinks of taking more than one room, except perhaps a few "bloated aristocrats" from U.S.A. For this one room he pays from 20 to 30 guldens a month, 12 guldens being about equal to £1, and in it he sleeps and generally breakfasts; but from eight to nine o'clock in the morning till bedtime he seldom visits it. Having secured a room, then, and endeavoured to give it a home-like air with his photos and books, he sallies out to the great hospital, the Allgemeine Krankenhaus, which contains about 2,000 beds. In the gateway are posted up notices giving particulars of the various courses of lectures given by the professors and their assistants. For instance, he may see that Herr Professor Doctor Adam Politzer will give a course of lectures on Diseases of the Ear, beginning on a certain day and lasting for six weeks—five days a week—at twelve o'clock, in his wards, in Hof. 2 (the second courtyard of the hospital); fee for the course, 20 guldens. The fees for lectures by professors are fixed by the University authorities; but the assistants, or privat-docenten, seem to charge at their own sweet will as much as they think they can get.

These courses are very numerous, and, as fresh ones are beginning each week, the new arrival has no difficulty in getting to work in a few days, entering for such courses as suit him. For instance, there are at least half a dozen privat-docenten, besides three or four professors, constantly lecturing on the eye; and as some of these give several lectures at different hours of the day, the choice of courses is abundant.

The majority of these lectures are given in the hospital wards, but not all, for near the hospital is the "Poliklinik," an enormous extern department, where thousands of out-patients are treated yearly. The staff of the Poliklinik consists of a number of professors not yet promoted to the intern department at the Krankenhaus, and their assistants.

Now let us follow our friend into Professor Politzer's ward—it will serve as a sample. We enter a large well-lighted room, with a dozen beds or so. Most of the patients, however, are up strolling about the room, chatting with others—old patients, who, though discharged from hospital, come in at certain times for treatment. The ward is more cheerful than most in one respect, for it is adorned with pictures of great aural surgeons, among which one of the late Sir William Wilde, of Dublin, holds an honoured place. Twelve o'clock strikes, and about thirty or forty young doctors stream in, an interesting sight. Here is a tall cadaverous Californian, who secured his degree, his compatriots tell us, after one year's study; and endeavours to look as wise as possible, lest he should be suspected of slight ignorance on that account. Next him is a neat little Japanese, followed by two calm Bostonians, and a hirsute German, with a well scarred visage, the result of many duels. Then comes a serious Scotchman, talking broad Aberdeen to a comfortable, happy-looking Englishman, evidently a public-school boy, and behind them, two tall curly-headed Irishmen, chaffing "Sandy" and "The Duke," as they call their friends in front. Jews, Hungarians, Turks, and Russians, interspersed with Austrians, follow, and all take their seats as the professor begins his lecture. This lasts about half an hour, then the patients are examined. While this is going on the professor talks freely to every man in his own tongue, except to the Japanese and Turks. He certainly speaks most European languages, and tries Latin with you if you do not seem to follow him clearly. One o'clock strikes, and the crowd streams out, the Scotchman at once resuming his argument with the Englishman on free education, and the Irishman making grabs at the tame pigeons, which rise almost from one's feet.

Every hour of the day, from seven or eight o'clock in the morning till six in the evening, may be occupied with classes, and the average British student will attend six or seven courses a day. The general plan is to have breakfast in one's room—coffee, rolls, and eggs (cooked in various ways)—then spend the day at the Krankenhaus and Poliklinik, taking a mid-day meal in a restaurant in any spare hour, and after more lectures, returning to the same restaurant for a substantial evening meal about six or seven. The favourite resort of the British and Americans for meals is the Riedhof; and among old Vienna students, Charlie, the head waiter there, is as widely known as anyone in the city, not even excepting Professor Billroth.

Following our friend inside the doors, we find large rooms furnished in much the same way as a London restaurant; but the food is very different. Charlie soon comes to us, presents the bill of fare, and helps us to choose our dishes, chatting away in very curious English—picked up, I believe, at an eating-house in the Strand. At first there is all the charm of novelty about the Wiener cookery, but that soon wears off, and one longs for a good substantial steak or "cut off the joint," and detests these endless made-up and "garnirt" dishes. One thing is most extraordinary, the omnipresence of the carraway, so unassuming at home. They even—may St. Patrick forgive them—sprinkle it plentifully over our potatoes, and we have to give special instructions to exclude it. The sweets, however, are excellent; and the "Schaumtorte"—whipped cream and chocolate held together by a slight framework of cake—is a thing to be remembered.

After the evening meal most men adjourn to a café, and one has one's regular café, just as at home one may have one's club. The best of those near the University take a number of English papers. In the Café International, for instance, in the Universitats Strasse, one finds the *Times*, *Standard*, *Scotsman*, *Punch*, *Graphic*, and *Lancet*. There are also generally a number of billiard tables, on which the cannon game is played; and as only about 6d. an hour is charged they are extensively patronised. Here a large proportion of the evenings are spent—reading, playing billiards, and chatting with one's friends; for little or no professional reading is done after such a long day's work at classes, and having only one room a man prefers not to go to it till he wishes to turn in.

It may be noted as a curious circumstance that Scotchmen are the last to leave the cafes and go home. The reason of this is that everyone going in after ten o'clock has to pay ten kreuzers (2d.) to the Hausemeister, and the Scotchman wants value for his money. The houses in Vienna are built on the same plan as those in Paris, and let in flats. At ten p.m. all lights are turned out in the passages and the house door shut; and everyone entering or leaving the house after that hour must ring for the hausemeister to open the door. Each person so doing must pay 10 kreuzers as he passes through, the only exception being that a husband and wife together count as one and pay accordingly. It is a most annoying tax, and though seemingly trivial, mounts up to a good sum per month; and the only way to regard it with equanimity is to look on it as an addition of a shilling or one and six a week to one's rent. One soon looks on the hausemeister as a natural enemy, and feels a glow of satisfaction when, having brought him from his bed in the small hours of the morning, with the thermometer near zero, one pays him the same coin as he would get at five minutes past ten.

If the student, feeling oppressed with the monotony of his work seeks variety, he has a large choice of places of amusement. The Opera and Hofburg Theatre are, of course, of world-wide reputation; but they are only patronised to a small extent by the young medical men, as they are decidedly expensive. A rather favourite resort for those who care for music is the Harmonie Saal, a large hall furnished as a restaurant with a platform at one end, where a band performs every evening. If the band is a regimental one, as is usually the case, the charge for admission is about sixpence, but if Strauss, one shilling. Of course one is expected to order refreshments, which may be only coffee or beer, but most visitors take the evening meal there, as the cooking is excellent and the prices moderate.

When there is frost—as there generally is for at least some weeks every winter—the Eislauf-verein is in great favour. It is a club possessing a magnificent rink, which is lighted by electric lamps at night, and enlivened by a regimental band every afternoon or evening. Doctors studying at the University are admitted without entrance fee, on payment of an annual subscription of ten guldens. Here many men hurry as soon as their classes are over for the day, and skate till late in the evening. There is a café in connection with the rink, into which one can easily scramble without taking off one's skates, so there is no necessity to pause long for supper. The event of the year here is the Ice Carnival, which is held, weather permitting, some time in January. Every skater must wear fancy costume, and as they number often over a thousand, the scene is a very brilliant one. It is said to be only surpassed by those at St. Petersburg and Montreal.

When the weather does not permit of skating, walking parties are much the fashion, especially in the holidays, which are very frequent, for Vienna was blessed with an enormous number of saints in days gone by. (The race seems to have died out now.) On these saints' days parties are made up by British or American residents, and the Wiener-wald, or wild-wooded hill country rising up from the Danube behind Vienna, is explored. Excursions are also made partly by rail and partly on foot to Greifenstein, an old castle on the Danube, and to Heiligin Kreuz, and other places of interest. In the clear, keen mountain air of the Wiener-wald it is no uncommon thing for English ladies to walk twenty or twenty-five miles, who at home would be tired out by half the distance. The paths over the wooded hills are marked out on an excellent system by the "Touristen Verein." This club or society publishes a map of the Wiener-wald, with the various footpaths marked on it in different colours. Suppose one wants to go over the Sofien Alpe to Hameau—a most lovely walk—one may find the path marked in red on the way, beginning at the roadside near some village. One easily finds one's way to the village, and as easily discovers the beginning of the path, marked with a red finger-post, "To the Sofien Alpe." Then, proceeding along the path, one finds at intervals the trees on each side marked with splashes of red paint. In this way a perfect stranger can find his way to all the best parts of the country within walking distance of Vienna. The whole country seems open to pedestrians, and makes one blush to think of the penalties attached to a walk on many a hill in our own free and enlightened land!

So with a good deal of work and some play, a winter in Vienna soon passes; and at its close Dr. Sawyer returns to his native land, benefited not only in his professional abilities, but in breadth of mind and knowledge of men and manners, all of which combine to make him a successful practitioner.

